# Resetting the clock on arthritis

**DOI:** 10.1186/s13075-019-1832-z

**Published:** 2019-01-29

**Authors:** Roman J. Krawetz

**Affiliations:** 1 McCaig Institute for Bone and Joint Health, University of Calgary, 3330 Hospital Drive NW, Calgary, Alberta T2N 4N1 Canada; 2 Department of Anatomy and Cell Biology, University of Calgary, Calgary, Alberta Canada; 3 Department of Surgery, University of Calgary, Calgary, Alberta Canada

## Abstract

While many of intricacies of the mammalian circadian rhythm remain unknown, the role that this physiological clock plays in our everyday lives and within the global ecosystem is clearly evident. However, only recently has the importance of circadian rhythm in cartilage health and joint homeostasis been brought to light. A recent study by Hand and colleagues advances our understanding of these processes further by demonstrating that disruption of circadian clock regulation in mesenchymal cells not only has an impact on the development of joint structures, but on the inflammatory response and on the onset/pathogenesis of arthritis.

In a recent issue of *Arthritis Research & Therapy*, Hand and colleagues [1] employed a transgenic mouse approach to increase our understanding of the role of the circadian clock within mesodermal-derived cells of the joint. The circadian clock regulates physiological responses in the body in response to ~ 24 h cycles present in our environment [2], instigated through patterns of light and temperature controlled by the rotation of the earth. It is important, however, to accept that the circadian clock is much more than an internal alarm clock that controls our wake/sleep cycles. It is now clear that this clock regulates daily cycles in the metabolic, immune, and endocrine systems, among others [3]. At the molecular level, the circadian clock is controlled by highly evolutionarily conserved genes, including both activators (Clock and Bmal1) and repressors (Per1/2 and Cry1/2) [2]. Disruption of circadian clock rhythm, through the aging process or intentional behaviors (e.g., working the night shift), has been positively correlated with the onset and pathogenesis of a number of human diseases [2, 4].

In specific regard to arthritis, it is not uncommon for patients suffering from arthritis (osteoarthritis (OA) and/or rheumatoid arthritis (RA)) to report cyclic patterns in pain and stiffness. Typically, an association is observed between increased pain during the night which keeps them awake, or increased stiffness in the morning [5]. While the exact mechanisms by which this occurs remains elusive, it has been suggested that the circadian clock differentially regulates inflammatory processes in relation to the wake/sleep cycle [6]. Transgenic mouse models have begun to shed light on the role of circadian clock rhythm in bone and joint development, maturation, and homeostasis, as mice with deficient clock regulation display abnormal joint morphology and increased susceptibility to inflammatory arthritis [7, 8]. Furthermore, isolated chondrocytes exposed to pro-inflammatory cytokines commonly observed during the arthritic disease process (e.g., IL-1β, TNFα) demonstrate disrupted clock gene expression [9]. These results, among others, have suggested an intimate relationship between circadian clock rhythm, inflammation, and arthritis.

While the chondrocytes that make up articular cartilage are targets of the arthritic disease process, fibroblast-like synoviocytes (FLS) present in the synovial membrane also play an essential role in the onset and propagation of pro-inflammatory cascades. These cascades contribute to articular cartilage degeneration and loss of articular chondrocytes. The role of circadian clock regulation in FLS has remained elusive. A study by Haas et al. demonstrated that FLS from OA and RA did express clock genes, but their expression was arrhythmic [10]. Hand and colleagues, however, were able to implicate FLS circadian rhythm in disease onset/progression by showing that FLS cells are indeed rhythmic and that the loss of circadian clock rhythm not only impacts their ability to regulate the inflammatory response, but also leads to worse disease progression in a model of arthritis [1]. However, given that Bmal1 was inactivated in all Col6a1-expressing cells throughout the development of the animal, it remains difficult to dissect what proportion of the observed effects were due to the loss of Bmal1 during developmental processes vs. post-induction of arthritis. Future studies where Bmal1 is inactivated in adult FLS could shed further light on the role of the circadian clock during the disease process specifically.

In their current study, Hand and colleagues also suggested that loss of Bmal1 may regulate osteochondral differentiation of synovial cells in vivo. Since it has previously been shown that synovial mesenchymal stem cells (MSCs) express Collagen type VI, it is possible that Col6a1-cre also inactivates Bmal1 in MSCs. To determine if Bmal1 and/or circadian clock regulation in general does affect FLS/MSC chondrogenic differentiation capacity (and therefore potential endogenous repair capacity within the joint), further studies directly focused on this topic should be undertaken.

The hypothesis that circadian rhythm may regulate stem cell behavior; and ultimately, endogenous repair is intriguing. Furthermore, Hand and colleagues’ study will direct future research into the role of the circadian clock (specifically Bmal1) in the regulation of local and systemic inflammatory responses. Both autocrine and paracrine pathways may be affected, and these signaling cascades could impact the overall health of the joint. It remains unknown if regulating the circadian clock in patients suffering from arthritis has any beneficial effect on clinical outcomes. However, the increasing number of studies implicating circadian rhythm in arthritis and/or cartilage health poses the question of whether interventions targeting circadian function (light, sleep, dietary, or physiological therapies) should be explored in arthritis patients. As our understanding of these multifactorial diseases known collectively as arthritis improves, so will our ability to treat these patients.
